# Editorial: Myocardial fibrosis: What we know now

**DOI:** 10.3389/fcvm.2022.1077070

**Published:** 2023-01-04

**Authors:** Gabriela Kania, Diana Lindner, Christian Zuppinger

**Affiliations:** ^1^Department of Rheumatology, Center of Experimental Rheumatology, University Hospital Zürich, University of Zurich, Zürich, Switzerland; ^2^Department of Cardiology and Angiology, Faculty of Medicine, University Heart Center Freiburg-Bad Krozingen, University of Freiburg, Freiburg im Breisgau, Germany; ^3^Department of Biomedical Research and Cardiology, Bern University Hospital, Bern, Switzerland

**Keywords:** myocardial fibrosis and inflammation, fibroblasts, macrophages, cell-to-cell contact, mechanism myocardial fibrosis, myocardial remodeling and extracellular matrix proteins, animal models of heart failure, 3D cell culture

Worldwide, cardiovascular diseases affect millions, cause serious economic burdens, and represent the number one cause of death. A broad range of pathological cardiac conditions is associated with myocardial tissue remodeling and fibrosis. Cardiac fibrosis reflects the exaggerated accumulation of extracellular matrix components, and activation of stromal cell compartments in the tissue, followed by acute or chronic inflammatory responses (see the Figure 1). Progressive cardiac fibrosis has been recognized to cause life-threatening arrhythmias. The development of life-saving therapeutic strategies and new medications requires extensive scientific efforts to understand the pathophysiology of pro-arrhythmogenic fibrosis, which is currently poorly understood. Understanding the cellular and molecular roots of cardiac fibrogenesis is crucial for identifying potential diagnostic and therapeutic targets in cardiovascular diseases. The aim of this Research Topic is a wide-ranging overview of the current understanding of the mechanisms of myocardial fibrosis across diverse cardiovascular disorders and its evaluation in patients.

**Figure 1:**
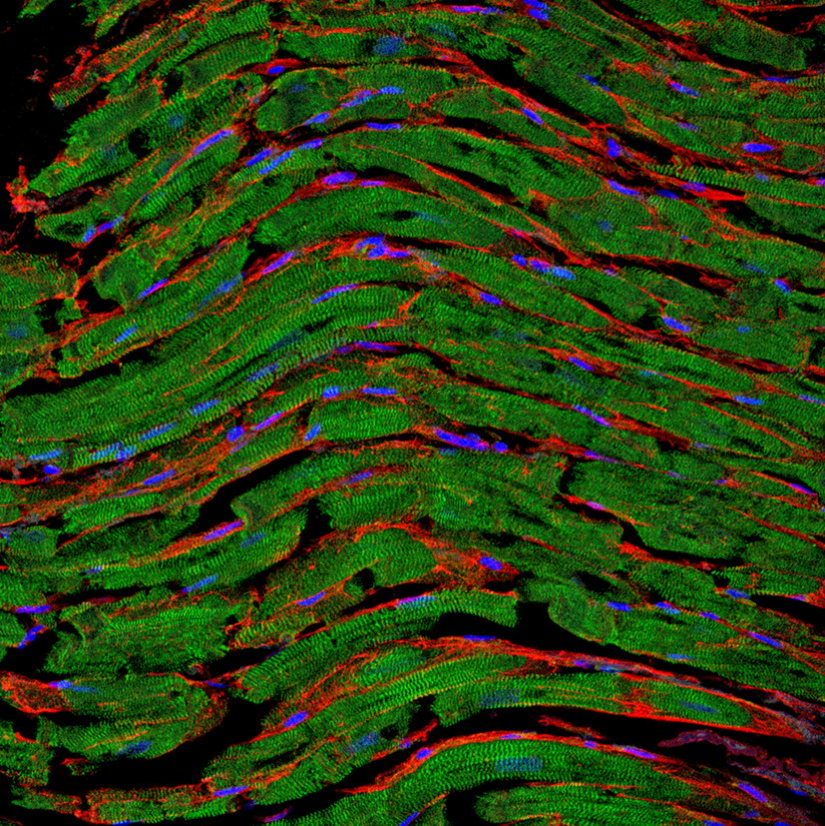
Representative fluorescence staining of paraffin-embedded cardiac tissue section derived from an autopsy patient. The clear sarcomere structure of cardiomyocytes is shown in green (α-actinin staining), nuclei are shown in blue (DAPI staining), and the fibrotic tissue between cells is highlighted in red (WGA staining). Image was kindly provided by Hanna Bräuninger.

*Review: Detection of myocardial fibrosis: where we stand*.

In the review article by Zhu et al., the authors provide a comprehensive overview of the current state of non-invasive detection methods for myocardial fibrosis with a focus on different techniques and clinical applications of cardiovascular magnetic resonance (CMR). Although new laboratory biomarkers are currently being explored, such as pro-collagens, galectin-3, or several miRNAs, CMR still is considered the non-invasive gold standard for providing information for the diagnosis, prognosis, risk stratification, and treatment of myocardial fibrosis.

*Original research: A clinical study on the relationship between the visualization of cardiac fibroblasts activation protein activity by Al18F-NOTA-FAPI-04 positron emission tomography and cardiovascular disease*.

Newly developed methods in positron emission tomography (PET) are discussed in the contribution by Lyu et al.. This particular technique makes use of a drug that binds to fibroblast activation protein (FAP) as a radiolabeled tracer to detect fibroblast activation in pathological conditions such as wound healing and cancer or, as highlighted in this study, the degree of fibrosis of coronary artery plaques. The authors conclude that the method is helpful for the early intervention and treatment of patients at elevated risk for cardiovascular disease, especially in patients with diabetes, obesity, and in the elderly.

*Original research: Fibulin-3 deficiency protects against myocardial injury following ischemia/reperfusion in vitro cardiac spheroids*.

An innovative experimental *in vitro* model, contracting spheroids made of primary neonatal cardiac cells from wild-type and KO-mice, is employed in the contribution by Sharma et al. to investigate the role of the extracellular matrix protein Fibulin-3 in myocardial injury and ischemia/reperfusion. The authors conclude that Fibulin-3 deficiency is protective against I/R injury in this 3D *in vitro* model.

*Original research: Zymosan A downregulates TGF-B1/Smad3 signaling to inhibit myocardial fibrosis after myocardial infarction*.

Tian et al. use Zymosan A, an activator of the innate immune response, in an experimental rat model of myocardial infarction to investigate the outcome of such an intervention on signaling pathways, gene expression, and ultimately fibrosis. It was found that early injection of Zymosan A at the edge of the infarcted area improves cardiac function and inhibits fibrosis mainly through local activation of macrophage aggregation, downregulation of TGF-beta1, upregulation of IL6, Connexin-43, and Discoidin Domain Receptor 2 (1).

*Original research: Characterize the differentiation process of myocardial fibroblasts under pressure overload and identify the markers that regulate myocardial fibrosis: A Bioinformatic Research of scRNA-seq Data*.

In the contribution by Li et al. a dataset obtained by single-cell RNA-seq of mice subjected to transverse aortic constriction, and of corresponding sham-operated animals, was analyzed with the goal of studying the heterogeneity of cardiac fibroblasts and their developmental trajectory. The authors found that fibroblast switched their metabolism from fatty acid oxidation to glycolysis during the transition to myofibroblasts. Additionally, changes in collagen synthesis, antioxidant gene expression, and angiogenesis were observed. Gstm1 was one of the most significantly down-regulated genes in the TAC-heart and might represent an anti-fibrotic factor (2).

*Study protocol: Effect of High-Intensity interval training, moderate continuous training, or guideline-based physical activity on peak oxygen uptake and myocardial fibrosis in patients with myocardial infarction: protocol for a randomized controlled trial*.

Shi et al. discuss high-intensity interval training (HIIT) for cardiac rehabilitation and propose a protocol for a clinical trial in patients with myocardial infarction. They hypothesize that peak oxygen uptake and myocardial fibrosis will be improved by high and moderate-intensity training.

*Original research: Myocardial extracellular volume fraction measured by cardiac magnetic resonance imaging negatively correlates with cardiomyocyte breath in a healthy porcine model*.

Non-invasive detection of the myocardial extracellular volume fraction (ECV) by magnetic resonance imaging is often used for estimating myocardial fibrosis, but this parameter varies greatly in healthy myocardium. Therefore, Zhang et al. studied ECV and cardiomyocyte breadth in tissue sections of healthy pig hearts to evaluate cellular geometry as an additional parameter in diagnosing early pathological changes in cardiovascular diseases. A negative correlation between ECV and cardiomyocyte breath was found in this model. Cellular morphologies in different cardiovascular diseases are discussed.

*Perspective: The role of cardiovascular magnetic resonance imaging in the assessment of myocardial fibrosis in young and veteran athletes: insights from a meta-analysis*.

In the contribution by Androulakis et al. the results of a meta-analysis are presented which was done to elucidate the incidence and appropriate methods for the assessment of myocardial fibrosis in athletes compared to healthy sedentary controls. They have found that CMR-analysis using non-specific late gadolinium enhancement and native T1 is useful in the discrimination of myocardial fibrosis in athletic vs. sedentary individuals.

*Original research: Echocardiographic global longitudinal strain is associated with myocardial fibrosis and predicts outcomes in aortic stenosis*.

Le et al. studied a cohort of hypertensive patients who underwent both CMR and echocardiography. They investigated the value of left ventricular global longitudinal strain (LV-GLS) thresholds for the risk-stratification of aortic stenosis patients with preserved left ventricular ejection fraction for myocardial replacement fibrosis.

## Author contributions

All authors listed have made a substantial, direct, and intellectual contribution to the work and approved it for publication.

## References

[1] Tian J Su G Sun P Xu X Qu S Chen J. Zymosan A downregulates TGF-β1 / Smad3 signaling to inhibit myocardial fibrosis after myocardial infarction. Front Cardiovasc Med. (2022).

[2] Li G Cheng Z Cheng C Wang R Luo X Qin Y. Characterization of the differentiation process of myocardial fibroblasts under pressure overload and identification of the markers that regulate myocardial fibrosis: a bioinformatic research of scRNA-seq data. Front Cardiovasc Med. (2022) 2022:3235250. 10.1155/2022/3235250 35799890 PMC9256463

